# Characterization of heavy users of skin care products among Norwegian women from 2003 to 2011

**DOI:** 10.1186/s13690-016-0165-5

**Published:** 2016-12-19

**Authors:** Boel Aniansson, Marit B. Veierød, Charlotta Rylander, Eiliv Lund, Torkjel M. Sandanger

**Affiliations:** 1 Department of Community Medicine, UiT-The Arctic University of Norway, Tromsø, Norway; 2 Department of Biostatistics, Oslo Centre for Biostatistics and Epidemiology, Institute of Basic Medical Sciences, Faculty of Medicine, University of Oslo, Oslo, Norway; 3 NILU, FRAM-High North Research Centre for Climate and Environment, Tromsø, Norway

**Keywords:** Skin care products, Daily use, Life style, Endocrine disruptors, Human exposure

## Abstract

**Background:**

Several ingredients in personal care products (PCPs) have been classified as endocrine disruptors (EDs) and concern has been raised whether use of PCPs can affect human health. We aimed to characterize Norwegian female users of skin care products and compare life style characteristics between heavy users and non-users.

**Methods:**

This cross-sectional study consisted of 114 202 women (age 41–76 years) participating in the Norwegian Women and Cancer study, a large population-based prospective cohort study. Based on self-reported questionnaire data, we classified the study subjects into five groups according to a calculated percentage of skin area creamed per day. Life-style characteristics were then compared between heavy users (using body lotion twice a day) and non-users. Change in use from 2003 to 2011 was assessed through repeated measurements (*n* = 8484).

**Results:**

Heavy users of skin care products had a significantly lower BMI, higher level of physical activity, higher income, higher alcohol consumption, fewer children and a shorter total time of breastfeeding than non-users (*p* < 0.001). There were significantly fewer current smokers and more former smokers among heavy users than among non-users (*p* < 0.01). Current and earlier use of oral contraceptives, hormone replacement therapy or hormonal intra-uterine device were significantly more common among heavy users than among non-users (*p* < 0.01). The use of skin care products was, to a moderate extent (weighted kappa 0.52), consistent over time from 2003 to 2011, and as many as 91.5% of the women were classified into the same user group ±1 category in 2003 and 2011.

**Conclusions:**

Heavy users of skin care products differ significantly from non-users on BMI, degree of daily physical activity, income, number of children, use of hormonal pharmaceuticals, smoking and alcohol consumption. Use of skin care products is common among women aged 41–76 years in Norway, and the use is consistent over time.

**Electronic supplementary material:**

The online version of this article (doi:10.1186/s13690-016-0165-5) contains supplementary material, which is available to authorized users.

## Background

The use of personal care products (PCPs), such as skin care products, deodorant and tooth paste are currently a part of many people’s daily hygiene routine. Some of the ingredients in PCPs have been identified as endocrine disruptors (EDs), i.e. molecules that are able to affect and interfere with the hormonal system of humans or wildlife. After dermal application of PCPs, possible EDs are easily absorbed by the epidermis into the subcutaneous tissue and the central blood circulation and concern has therefore been raised whether the use of PCPs can affect human health.

Of possible EDs in PCPs, frequently mentioned are ultraviolet-filters (UV-filters), triclosan, phthalates and parabens [1]. Some of the UV-filters found in sunscreen products show endocrine disruptive activity in vitro and/or in vivo [2]. Triclosan has been a preservative in PCPs since the late 1960s, and has been detected in human fluids like urine, serum and breast milk [3]. Triclosan can bind to both the estrogen and the androgen receptor, but in vitro and in vivo consequences of this have been diverse [3]. Phthalates, also called plasticizers, are a group of molecules increasing the softness of plastics. Phthalates can appear in fragrances and hair products as fixatives [4], but also leak from packaging material into the product. Their endocrine disrupting mechanism has been suggested to be interference with testosterone production or the testosterone action [1]. Parabens have been one of the most frequently used preservatives in PCPs since the beginning of the 20th century [5]. Parabens are able to bind to the estrogen receptor [6], and the affinity increases with the length of the alkyl chain. Long chained parabens (isopropylparaben, isobutylparaben, phenylparaben, benzylparaben and pentylparaben) have been banned in PCPs within the European Union (EU) during the last years, and the concentrations for propyl- and butylparaben in PCPs have recently been reduced [7].

In our recent study [8], we found elevated concentrations of methylparaben (MP) in blood samples from Norwegian women frequently using skin care products (defined as creams and lotions for skin care), indicating that everyday use may keep the concentration of parabens continuously high in blood, despite a short half-life. A recent Swedish study found a correlation between urinary concentrations of parabens and the use of PCPs, and a US study found a correlation between urinary concentrations of some phenols and phthalate metabolites and the use of PCPs in both adults and children, suggesting that the use of PCPs is an important source of exposure to several EDs [4, 9].

PCPs often contain a complex mixture of EDs, and as there is limited knowledge about the collective effect of these chemicals on human health, population studies addressing the association between PCP use and hormonally related diseases are needed. As a first step in this process, it is important to characterize the heavy users of these products.

Between 2003 and 2011, more than 100 000 women in the Norwegian Women and Cancer (NOWAC) study answered questionnaires covering a large number of lifestyle factors including the use of skin care products. The aim of this study was to describe and compare lifestyle characteristics between heavy users of skin care products and non-users. To the best of our knowledge, published studies on use of skin care products have not involved a sample of this size, nor a characterization of the participants regarding life style characteristics and endocrine events in a woman’s life such as menarche, pregnancy, breastfeeding and menopause, which are important factors for hormonally related diseases.

## Methods

### Study cohort

NOWAC is a population based prospective cohort study, initiated in 1991 with the aim of investigating the etiology of different cancers among Norwegian women [10]. More than 170 000 Norwegian women aged 30–70 years when entering the study have been enrolled in the study so far. The participating women are randomly chosen from the National Population Registry in Norway, and invited to participate in the survey through an invitation letter sent to their home address. All participants have answered a detailed questionnaire on current health status, diet, medication use, physical activity and other lifestyle habits at enrollment. The questionnaires have been distributed in series and the questions in the different series have varied a little over years. The external validity of the NOWAC study has been thoroughly evaluated and found satisfactory and without selection bias [11].

### Assessment of use of skin care products

In the distributed questionnaires, the participants were asked how often they use skin care products named as body lotion, hand cream and facial cream (never/seldom, 1–3 times per month, once a week, 2–4 times per week, 5–6 times per week, 1 time/day and 2 or more times/day). We converted the reported frequency of a certain cream type into a percentage of body surface covered in cream. Use of body lotion once per day was set equivalent to 91% of the body surface covered in cream, hand cream 6% and facial cream 3% (Additional file 1: Table S1). When the use of each of these was reported once per day, 100% of the body surface was covered in cream. Further, using body lotion once per week corresponds to a seventh of 91%, i.e., 13% of the body surface covered in cream per day. These conversions were similar to our previous article [8]. Total body area creamed per day was calculated as the sum of the percentage surface area covered by body lotion, hand cream and facial cream. If a study subject had answered at least one question on use of skin care products, a missing value on the other cream or lotion questions was interpreted as “never/seldom” (*n* = 1776). By this procedure, we could have underestimated the use of skin care products for these women, however, running the analyses without these subjects did not change the results and they were therefore included.

### Assessment of life style characteristics

The age of the woman at the time of filling in the questionnaire was extracted from the National Population Registry. The women were asked to report their current weight (kg) and height (cm) in the questionnaire, and body mass index (BMI, kg/m^2^) was calculated. The women were also asked how many years of education they had in total and the yearly income of their household. Overall current physical activity level (including both recreational and occupational activity) was recorded on a rating scale from 1 (very low) to 10 (very high), which has been validated against a combined heart rate and movement sensor according to the European Prospective Investigation into Cancer and Nutrition protocol [12]. Questions about smoking history and current smoking were combined into a variable for smoking status (never, former and current). The participants were asked whether they were absolutists or how often they drink different types of alcohol (beer, wine or strong spirits); never/seldom, 1–3 times/month, 1 time/week, 2–4 times/week, 5–6 times/week, 1 time/day and ≥2 times/day). The recorded frequency was later converted to a combined equivalent of alcohol consumption in grams of pure alcohol per day, described in more detail by Dumeaux et al. [13]. Age at first birth was calculated from the birth year of the woman and the birth year of the child, which was requested in the questionnaire. The women were asked about how many children they had, and how many months they had been breastfeeding throughout their life-course. Age at menarche and menopause was also inquired. Questions on use of oral contraceptives (OC), hormone replacement therapy (HRT) and hormone releasing intrauterine device (IUD) was included in the questionnaire (current use, former use and never used). Sunscreen use in Norway, during vacation in southern latitudes and at Easter holiday was recorded, as well as showering with and without soap (never/seldom, 2–3 times/month, 1 time/week, 2–3 times/week, 4–6 times/week, 1 time/day and >1 time/day). Perfume use was also recorded (never/seldom, 2–3 times/month, 1 time/week, 2–3 times/week, 4–6 times/week, 1 time/day and >1 time/day).

### Study sample

In total, 114 794 women have returned the questionnaires that included questions on skin care product use between 2003 and 2011 (205 519 invited, 55.9% response rate). After excluding participants with missing values for all three cream questions (body lotion, hand cream and facial cream) (*n* = 569), age of birth <12 years (*n* = 8), age at menarche ≥30 years (*n* = 3) and age at menopause <16 years (*n* = 12), there were 114 202 women included in the analysis of skin care product use. Of the women answering the questionnaire in 2003, 8899 women have also filled in a follow-up questionnaire in 2011, where 8484 had answered at least one question on use of products for skin care.

### Statistical methods

Descriptive data is presented as frequencies, percentages, means with standard deviations (SDs) and medians with 25^th^ and 75^th^ percentiles. Statistical assessment of differences between user groups was carried out using one-way analysis of variance (ANOVA) or Kruskal-Wallis test for continuous variables, and chi-square test for categorical variables. When overall significant differences were found, comparisons between individual groups were carried out with t-test, Mann-Whitney test or chi-squared test, respectively. We analyzed trends across categories of a variable by assigning equally spaced values (1, 2, 3, 4 and 5) to user groups of skin care products and treating the variable as a continuous variable in a linear regression analysis. Pearson’s correlation coefficient (r_P_) was calculated for some variables. In the sub-sample of women with repeated information on use of skin care products, we studied change over time (from 2003 to 2011). From a two-way contingency table of the two responses, we present the proportion of women on the diagonal (absolute agreement) and on the diagonal ±1 category as P_A_ and P_A ±1_, respectively. The symmetry was assessed by calculating the proportion of study subjects with second assessment category being higher than the first assessment category. Weighted kappa (κ_w_) was also estimated (Cicchetti–Allison weights). STATA (version 13.0, StataCorp LP, College Station, Texas, USA) was used for the statistical analyses.

## Results

The mean age of the 114 202 women was 54.8 years (range 41–76 years), and they were born between 1928 and 1963 (mean 1950). In total, 26.7% of the women used body lotion at least once per day (Table 1). Hand cream use was more common, with 52.2% reporting use once a day or more. Facial cream was by far the most frequently applied cream, used daily or more by 82.9% of the women.

**Table 1:** Use of skin care products in the study sample (*n* = 114 202). The NOWAC study, 2003–2011, Tromsø, Norway

	Body lotion n (%)	Hand cream n (%)	Facial cream n (%)
Never/seldom	15615 (14.1)	12671 (11.5)	6816 (6.0)
1–3 times/month	11974 (10.8)	8723 (7.9)	2555 (2.3)
1 time/week	14602 (13.2)	7640 (6.9)	1797 (1.6)
2–4 times/week	28455 (25.6)	15707 (14.3)	4598 (4.1)
5–6 times/week	10697 (9.6)	7983 (7.2)	3547 (3.1)
1/day	27343 (24.6)	25680 (23.3)	65270 (57.7)
≥2/day	2340 (2.1)	31840 (28.9)	28508 (25.2)
Total	110996 (100)	110244 (100)	113091 (100)
Missing values	3206	3958	1111

Total percentage of body surface creamed every day ranged from non-users of skin care products to women creaming an equivalent of 200% of their body surface every day (Table 2). Use of body lotion twice a day yields a percentage of 182% (Additional file 1: Table S1). Based on their calculated percentage of body surface creamed per day, the women were categorized into five groups: non users (0% body surface creamed per day, *n* = 2131), light users (0.001–34.99%, *n* = 43 239), moderate users (35–64.99%, *n* = 28 455), frequent users (65–114.99%, *n* = 38 037) and heavy users (115–200%, *n* = 2340). There were no observations with a total percentage of creamed body surfaces between 115 and 182%, so all women characterized as heavy users of skin care products used body lotion twice a day (Additional file 1: Figure S1 and Table 2). Of the heavy users, 77.0% used hand cream and 85.7% used face cream twice a day (Table 2).

**Table 2:** Frequency (%) of skin care product use in the five different user groups (*n* = 114 202). The NOWAC study, 2003–2011, Tromsø, Norway

	Use of skin care products				
	None	Light	Moderate	Frequent	Heavy
Body surface creamed/day^a^ (%)	0	0.001–34.99	35–64.99	65–114.99	115–200
	n (%)	n (%)	n (%)	n (%)	n (%)
Total	2131 (1.9)	43239 (37.9)	28455 (24.9)	38037 (33.3)	2340 (2.1)
Body lotion					
Never/seldom	2027 (100)	13588 (33.8)	0	0	0
1–3/month	0	11947 (29.8)	0	0	0
1/week	0	14602 (36.4)	0	0	0
2–4/week	0	0	28455 (100)	0	0
5–6/week	0	0	0	10694 (28.1)	0
1/day	0	0	0	27343 (71.9)	0
≥2/day	0	0	0	0	2340 (100)
Hand cream					
Never/seldom	2033 (100)	6593 (15.9)	2022 (7.3)	1938 (5.3)	85 (3.8)
1–3/month	0	6064 (14.7)	1460 (5.3)	1164 (3.2)	35 (1.6)
1/week	0	4552 (11.0)	1776 (6.4)	1272 (3.5)	40 (1.8)
2–4/week	0	6974 (16.9)	5324 (19.2)	3328 (9.0)	81 (3.6)
5–6/week	0	2785 (6.7)	2507 (9.1)	2640 (7.2)	51 (2.3)
1/day	0	7507 (18.1)	6456 (23.3)	11489 (31.1)	228 (10.1)
≥2/day	0	6902 (16.7)	8138 (29.4)	15062 (40.8)	1738 (77.0)
Face cream					
Never/seldom	2057 (100)	3532 (8.3)	606 (2.1)	582 (1.5)	39 (1.7)
1–3/month	0	2172 (5.1)	237 (0.8)	144 (0.4)	2 (0.1)
1/week	0	1383 (3.2)	270 (1.0)	140 (0.4)	4 (0.2)
2–4/week	0	2942 (6.9)	1325 (4.7)	319 (0.9)	12 (0.5)
5–6/week	0	1878 (4.4)	909 (3.2)	748 (2.0)	12 (0.5)
1/day	0	24424 (57.1)	17482 (61.9)	23105 (61.3)	259 (11.3)
≥2/day	0	6461 (15.1)	7428 (26.3)	12649 (33.6)	1970 (85.7)

^a^More than a 100% body surface creamed corresponds to creaming the body more than once a day (see text)

Among non-users, 39.9% reported sunscreen use in Norway, 38.3% in southern latitudes and 37.5% during Easter holiday, whereas in the more frequent user groups (frequent and heavy users), up to 79.0% of the women used sunscreen in these occasions (Table 3). Daily perfume use was more common among frequent and heavy users of skin care products (47.6 and 35.4%, respectively), than among non-users (16.6%). Showering with soap was more common among heavy users (median 30 times/month) than among non-users (median 10 times/month) (Table 3). Use of body lotion was moderately correlated to showering (r_P_ = 0.27, *p* < 0.01), and less to physical activity (r_P_ = 0.13, *p* < 0.01).

**Table 3:** Use of other personal care products in five different user groups of skin care products (*n* = 114 202). The NOWAC study, 2003–2011, Tromsø, Norway

	Use of skin care products				
	None	Light	Moderate	Frequent	Heavy
	n (%)	n (%)	n (%)	n (%)	n (%)
	2131 (100)	43239 (100)	28455 (100)	38037 (100)	2340 (100)
Sunscreen^a^					
In Norway	851 (39.9)	29714 (68.7)	22441 (78.9)	30287 (79.6)	1848 (79.0)
In southern latitudes	817 (38.3)	26790 (61.9)	20778 (73.0)	28671 (75.4)	1711 (73.1)
At Easter holiday	800 (37.5)	27305 (63.2)	20522 (72.1)	27978 (73.6)	1629 (69.6)
Perfume^b^					
Never/seldom	1089 (53.0)	11338 (27.0)	5210 (18.5)	5608 (15.0)	358 (15.8)
1–3/month	259 (12.6)	6072 (14.4)	3144 (11.2)	2884 (7.7)	159 (7.0)
1/week	133 (6.5)	3693 (8.8)	2598 (9.2)	2708 (7.24)	131 (5.8)
2–4/week	160 (7.8)	5654 (13.5)	4748 (16.9)	5080 (13.6)	218 (9.6)
5–6/week	67 (3.3)	2272 (5.4)	1891 (6.7)	2285 (6.1)	69 (3.0)
1/day	340 (16.6)	12523 (29.8)	10118 (36.0)	17783 (47.6)	805 (35.4)
≥2/day	7 (0.3)	492 (1.2)	427 (1.5)	1052 (2.8)	532 (23.4)
	Median (25^th^–75^th^ percentile)	Median (25^th^–75^th^ percentile)	Median (25^th^–75^th^ percentile)	Median (25^th^–75^th^ percentile)	Median (25^th^–75^th^ percentile)
Shower frequency^c^	10 (10–30)	20 (10–30)	10 (10–20)	30 (20–30)	30 (20–30)

^a^1 observation missing

^b^2295 observations missing

^c^2780 observations missing

Table 4 provides information on demographics and life style characteristics in the user groups. Use of skin care products was significantly associated with all investigated variables (*p* ≤ 0.01), except age at menarche (*p* = 0.36). For significant variables, we compared differences between non-users and heavy users. Heavy users of skin care products had significantly higher education, higher household income, higher level of physical activity and higher intake of alcohol than non-users (*p* < 0.01). Likewise, heavy users reported significantly lower BMI (mean difference: −3.47 kg/m^2^), less children and fewer months of total breastfeeding as compared to non-users (*p* < 0.01). Age at menopause did not differ significantly between heavy users and non-users (*p* = 0.56), neither did age at first birth (*p* = 0.58). There were significantly fewer current smokers and more former smokers among heavy users than among non-users (*p* < 0.01). Current or earlier use of OCs, HRT or IUD was significantly more common among heavy users than among non-users (*p* < 0.01). Likewise, comparison of non/light versus frequent/heavy users were significant for all these variables (*p* ≤ 0.01), except for use of hormone IUD at time of answering the questionnaire (*p* = 0.33).

**Table 4:** Demographics and life style characteristics in the five different user groups. The NOWAC study, 2003–2011, Tromsø, Norway

	Use of skin care products					
	None	Light	Moderate	Frequent	Heavy	Group comparison
Number of observations	2131	43239	28455	38037	2340	
Characteristics	Mean (SD)	Mean (SD)	Mean (SD)	Mean (SD)	Mean (SD)	*p*-value
Age at answering questionnaire	55.0 (5.0)	54.6 (4.8)	55.0 (4.9)	54.9 (4.8)	54.9 (4.8)	*p* < 0.01^a^
Education, years	12.3 (3.9)	13.1 (3.6)	12.7 (3.4)	12.9 (3.4)	13.0 (3.5)	*p* < 0.01^a^
Household income, 10 000’s NOK	47.9 (23.2)	55.9 (22.0)	56.7 (21.4)	58.3 (22.1)	56.1 (23.4)	*p* < 0.01^a^
BMI, kg/m^2^	27.7 (5.8)	25.7 (4.8)	25.1 (4.0)	24.7 (3.8)	24.2 (3.9)	*p* < 0.01^a^
Physical activity^d^	5.1 (2.1)	5.6 (1.8)	5.9 (1.8)	6.1 (1.8)	6.5 (2.0)	*p* < 0.01^a^
Age at menarche	13.2 (1.5)	13.3 (1.4)	13.3 (1.4)	13.3 (1.4)	13.2 (1.5)	*p* = 0.36^a^
Age at menopause	48.5 (5.2)	49.0 (4.8)	49.1 (4.7)	49.0 (4.9)	48.6 (5.3)	*p* < 0.01^a^
Age at first birth	23.9 (4.7)	24.3 (4.7)	24.0 (4.4)	24.0 (4.4)	23.8 (4.3)	*p* < 0.01^a^
Number of children	2.4 (1.4)	2.2 (1.2)	2.2 (1.1)	2.1 (1.1)	2.1 (1.1)	*p* < 0.01^a^
	Median (25^th^–75^th^percentile)	Median (25^th^–75^th^percentile)	Median (25^th^–75^th^percentile)	Median (25^th^–75^th^percentile)	Median (25^th^–75^th^percentile)	
Breast feeding, months	11 (4–21)	12 (6–21)	11 (5–19)	10 (5–18)	10 (4–19)	*p* < 0.01^b^
Alcohol, gr/day	0.95 (0–3.3)	1.98 (0.5–5.5)	2.55 (0.9–6.2)	2.92 (1.0–6.3)	2.53 (0.9–6.3)	*p* < 0.01^b^
	%	%	%	%	%	
Smoking status						
Never	39.9	38.8	35.0	34.0	34.4	*p* < 0.01^c^
Former	27.4	37.7	41.0	42.2	40.3	
Current	32.7	23.5	24.0	23.8	25.3	
Use of OC						
Never	48.4	31.3	38.2	36.0	36.6	*p* < 0.01^c^
Former	51.0	58.3	61.3	63.5	62.6	
Current	0.6	0.5	0.5	0.6	0.8	
Use of HRT						
Never	73.8	66.6	60.6	57.8	53.8	*p* < 0.01^c^
Former	17.0	20.7	23.6	23.4	24.4	
Current	9.2	12.7	15.8	18.8	21.8	
Use of Hormone IUD						
Ever	8.1	11.1	11.6	11.9	12.3	*p* < 0.01^c^ *p* < 0.01^c^
Current	5.9	10.7	10.7	10.8	9.9	

*BMI* body mass index, *OC* oral contraceptive, *HRT* hormone replacement therapy, *IUD* intra uterine device

^a^One-way analysis of variance, ^b^Kruskal-Wallis test, ^c^Chi-square test

^d^Daily physical activity, from 1 = very little to 10 = very much

Noteworthy, BMI decreased linearly with increased use of skin care products (p for trend <0.01), and physical activity increased linearly with increased cream use (p for trend <0.01).

Characteristics according to use of body lotion, hand cream and facial cream are provided in Additional file 1: Tables S2–S4. Moderate, frequent and heavy users of body lotion were the same individuals as those categorized as moderate, frequent and heavy users of skin care products (Additional file 1: Table S3). The same variables that characterize heavy users of skin care products and body lotion, also describe heavy users of facial cream (Additional file 1: Table S4), whereas hand cream users differed mainly in BMI, physical activity, smoking status and use of synthetic hormones (Additional file 1: Table S4).

### Comparison of cream use in 2003 and 2011

In total, 8484 women answered questions on cream use in 2003 and in 2011 (Table 5). A majority of the women reported use of skin care products so similar they were categorized in the same group both years (absolute agreement P_A_ = 59.9%, Table 6) and 91.5% are within ± 1 category (P_A±1_ = 91.5%), while 25.6% of the women recorded increased use and 14.5% decreased use in 2011. Absolute agreement was highest for facial cream (P_A_ = 66.6%) and lowest for hand cream (P_A_ = 40.6%). Likewise, weighted kappa was highest for facial cream (κ_w_ = 0.59) and lowest for hand cream (κ_w_ = 0.51) (Table 6).

**Table 5:** User group among women answering question both in 2003 and in 2011 (*n* = 8484). The NOWAC study, 2003–2011, Tromsø, Norway

Use of skin care products in 2003,	Use of skin care products in 2011, n (% of total)					
n (% of total)	None	Light	Moderate	Frequent	Heavy	Sum
None	58 (0.7)	55 (0.6)	8 (0.1)	8 (0.1)	0 (0.0)	129 (1.5)
Light	82 (1.0)	1879 (22.1)	728 (8.6)	490 (5.8)	14 (0.2)	3193 (37.6)
Moderate	6 (0.1)	363 (4.3)	969 (11.4)	743 (8.9)	18 (0.2)	2099 (24.7)
Frequent	2 (0.0)	157 (1.9)	496 (5.8)	2133 (25.1)	112 (1.3)	2900 (34.2)
Heavy	0 (0.0)	3 (0.0)	12 (0.01)	107 (1.3)	41 (0.5)	163 (1.9)
Sum	148 (1.7)	2457 (29.0)	2213 (26.1)	3481 (41.0)	185 (2.2)	8484 (100.0)

**Table 6:** Agreement between reported use of skin care products in 2003 and 2011. The NOWAC study, 2003–2011, Tromsø, Norway

	Number	P_A_ (%)^c^	P_A±1_ (%)^d^	κ_w_ (95% CI)^e^
Body lotion^a^	8485	44.4	75.0	0.52 (0.51–0.53)
Hand cream^a^	8408	40.6	70.9	0.51 (0.50–0.51)
Facial cream^a^	8740	66.6	90.3	0.59 (0.58–0.61)
User status^b^	8484	59.9	91.5	0.52 (0.51–0.53)

^a^Never/seldom, 1–3 times per month, once a week, 2–4 times per week, 5–6 times per week, 1 time/day and 2 or more times/day

^b^None, light, moderate, frequent, heavy

^c^Percentage of agreement on the diagonal

^d^Percentage of agreement on the diagonal ± 1 category

^e^Weighted kappa with 95% confidence interval (CI)

## Discussion

In this nationally representative study sample of more than 114 000 Norwegian women, we have assessed lifestyle characteristics in relation to use of skin care products. Our findings indicate that women classified as heavy users (using body lotions twice a day or more) have a distinctively different lifestyle than non-users, with higher income, higher level of physical activity, lower BMI, fewer children, higher alcohol consumption and a greater tendency to use hormones (OCs, HRT and IUD) during their life span. Smoking was also differently distributed – it was, to a larger extent, a former habit among heavy users compared to non-users, who were more often current smokers. The lack of difference in age at menarche and age at menopause is consistent with the impression that the differences mainly occur within characteristics that the women can affect themselves. Characteristics of heavy users of body lotion, facial cream and hand cream were mainly in agreement with the characteristics of heavy users of total skin care products.

The agreement between 2003 and 2011 was moderate (κ_w_ = 0.52) [14], and the habit of using skin care products is therefore clearly established among women aged 41–76 years in Norway. At what time in life creaming habits are established in Norway is not studied here, but other studies show that the use of skin care products is widespread among adolescents and children in other European countries [15, 16].

An important strength of this study is the large, population based study sample reflecting cream use among Norwegian women (41–76 years old) in recent time. To the best of our knowledge, this is the largest study conducted on use of skin care products in comparison to other life style factors. Limitations include no data on deodorant use, a cohort not including younger women and exposure classification based on self-reported data. However, it is previously shown [8] that self-reported use of skin care products is highly correlated with paraben concentrations in plasma samples from the same cohort and we therefore consider the risk of exposure misclassification as acceptable. As it is unclear whether dermal absorption of EDs on specific areas or continuously elevated concentrations of EDs in plasma is of importance for endocrine related diseases, our lack of ability to differentiate between frequent low exposure (e.g. daily application of facial cream) and less frequent higher exposure (e.g. use of body lotion once a week), when using the conversion table, can be seen upon as a weakness. However, based on our previous study [8], it is the calculated daily percentage of exposed skin area that determines plasma concentration for parabens, and our exposure classification is therefore a valid measure of systemic uptake.

## Conclusion

Heavy users of skin care products differ significantly from non-users on BMI, degree of daily physical activity, income, number of children, use of hormonal pharmaceuticals, smoking and alcohol consumption. Use of skin care products is common among women aged 41–76 years in Norway, and the use is consistent over time to a moderate extent.

## Additional file

Additional file 1: Table S1. Conversion table of type and frequency of skin care product into percentage body area creamed per day. The NOWAC study, 2003–2011, Tromsø, Norway. **Table S2.** Demographics and life style characteristics in the five different user groups of body lotion. The NOWAC study, 2003–2011, Tromsø, Norway. **Table S3.** Demographics and life style characteristics in the five different user groups of facial cream. The NOWAC study, 2003–2011, Tromsø, Norway. **Table S4.** Demographics and life style characteristics in the five different user groups of hand cream. The NOWAC study, 2003–2011, Tromsø, Norway. **Figure S1.** Distribution of the calculated total percentage of body surface creamed per day (*n* = 114 202). The NOWAC study, 2003–2011, Tromsø, Norway. (DOCX 208 kb)

## Abbreviations

ANOVA: Analysis of variance
BMI: Body mass index
EDs: Endocrine disruptor
HRT: Hormone replacement therapy
IUD: Hormone releasing intrauterine device
NOWAC: Norwegian women and cancer study
OCs: Oral contraceptives
P_A_ and P_A ±1_: Describes the symmetry in a two-way contingency table of repeated measurements, i.e. P_A_ is the proportion of participants with absolute agreement between the two measurements and P_A+1_ is the proportion of women with absolute agreement ±1 category
PCPs: Personal care products
r_p_: Person’s correlation coefficient
SD: Standard deviation
UV-filters: Ultraviolet filters
κ_w_: Weighted kappa
