# Supplementary material for: Characterization of heavy users of skin care products among Norwegian women from 2003 to 2011
Source: Arch Public Health. 2016 Dec 19;74:53. doi: 10.1186/s13690-016-0165-5 (PMC5165705; doi:10.1186/s13690-016-0165-5)

**Supplementary material to the manuscript entitled:**

Characterization of heavy users of skin care products among Norwegian women from 2003 to 2011.

Boel Aniansson^1^, Marit B Veierød^2^, Charlotta Rylander^1^, Eiliv Lund^1^, Torkjel M Sandanger^1, 3^

^1^ Department of Community Medicine, UiT- the Arctic University of Norway, Tromsø, Norway

^2^ Oslo Centre for Biostatistics and Epidemiology, Department of Biostatistics, Institute of Basic Medical Sciences, Faculty of Medicine, University of Oslo, Oslo, Norway

**^3^**NILU, FRAM-High North Research Centre for Climate and Environment, Tromsø, Norway

**Corresponding author:**

**Boel Aniansson,** Department of Community Medicine, UiT- the Arctic University of Norway, N-9010 Tromsø, Norway.

Email: boel.aniansson@gmail.com

**Table S.1.** Conversion table of type and frequency of skin care product into percentage body area creamed per day. The NOWAC study, 2003-2011, Tromsø, Norway.

|  | **Type of cream** | | |
| --- | --- | --- | --- |
| **Frequency** | **Body lotion** | **Hand cream** | **Facial cream** |
| Never/seldom | 0 | 0 | 0 |
| 1-3 times/month | 6.5 | 0.4 | 0.2 |
| 1 time/week | 13 | 0.8 | 0.4 |
| 2-4 times/week | 39 | 2.6 | 1.3 |
| 5-6 times/week | 71.5 | 4.8 | 2.4 |
| 1/day | 91 | 6 | 3 |
| ≥2/day | 182 | 12 | 6 |

**Supplementary table S2.** Demographics and life style characteristics in the five different user groups of body lotion. The NOWAC study, 2003-2011, Tromsø, Norway.

|  | **Use of body lotion** | | | | | |
| --- | --- | --- | --- | --- | --- | --- |
|  | **None** | **Light** | **Moderate** | **Frequent** | **Heavy** | **Group comparison** |
| Reported frequency of body lotion use | Never | 1-4 times/month | 2-4 times/week | 5-7 times/week | ≥2 times/day |  |
| Body surface creamed/day (%) | 0 | 6.5-13.0 | 39.0 | 71.5-91.0 | 182 | - |
| Number of Observations | 18821 | 26549 | 28455 | 38037 | 2340 | - |
| Characteristics | Mean (SD) | Mean (SD) | Mean (SD) | Mean (SD) | Mean (SD) | p-value |
| Age at answering questionnaire | 54.6 (4.9) | 54.5 (4.8) | 55.0 (4.9) | 54.9 (4.8) | 54.9 (4.8) | p<0.01^1^ |
| Education, years | 12.9 (3.8) | 13.1 (3.6) | 12.7 (3.4) | 12.9 (3.4) | 13.0 (3.5) | p<0.01^1^ |
| Household income, 10 000’s NOK | 53.7 (22.4) | 56.8 (21.8) | 56.7 (21.4) | 58.3 (22.1) | 56.1 (23.4) | p<0.01^1^ |
| BMI, kg/m^2^ | 26.2 (4.8) | 25.5 (4.2) | 25.1 (4.0) | 24.7 (3.8) | 24.2 (3.9) | p<0.01^1^ |
| Physical activity^4^ | 5.6 (1.9) | 5.7 (1.8) | 5.9 (1.8) | 6.1 (1.8) | 6.5 (2.0) | p<0.01^1^ |
| Age at menarche | 13.3 (1.4) | 13.3 (1.4) | 13.3 (1.4) | 13.3 (1.4) | 13.2 (1.5) | p<0.01^1^ |
| Age at menopause | 49.0 (4.9) | 49.0 (4.7) | 49.1 (4.7) | 49.0 (4.9) | 48.6 (5.3) | p<0.01^1^ |
| Age at first birth | 24.3 (4.8) | 24.3 (4.7) | 24.0 (4.4) | 24.0 (4.4) | 23.8 (4.3) | p<0.01^1^ |
| Number of children | 2.3 (1.3) | 2.2 (1.2) | 2.2 (1.1) | 2.1 (1.1) | 2.1 (1.1) | p<0.01^1^ |
|  | Median  (25^th^ – 75^th^ percentile) | Median  (25^th^ – 75^th^ percentile) | Median  (25^th^ – 75^th^ percentile) | Median  (25^th^ – 75^th^ percentile) | Median  (25^th^ – 75^th^ percentile) |  |
| Breastfeeding, months | 12 (5- 21) | 12 (6-20) | 11 (5-19) | 10 (5-18) | 10 (4-19) | p<0.01^2^ |
| Alcohol, gr/day | 1.5 (0-4.9) | 2.3 (0.8-5.9) | 2.6 (0.9-6.2) | 2.9 (1.0-6.3) | 2.5 (0.9-6.3) | p<0.01^2^ |
|  | % | % | % | % | % |  |
| Smoking status  Never  Former  Current | 40.5  35.9  23.6 | 37.7  38.2  24.1 | 35.0  41.0  24.0 | 34.0  42.2  23.8 | 34.4  40.3  25.3 | p<0.01^3^ |
| Use of OC  Never  Former  Current | 45.4  54.1  0.5 | 39.0  60.5  0.5 | 38.2  61.3  0.5 | 36.0  63.4  0.6 | 36.6  62.6  0.8 | p<0.01^3^ |
| Use of HRT  Never  Former  Current | 69.5  19.1  11.4 | 65.1  21.5  13.4 | 60.6  23.6  15.8 | 57.8  23.4  18.8 | 53.8  24.4  21.8 | p<0.01^3^ |
| Use of Hormone IUD  Ever  Current | 10.2  9.1 | 11.4  11.4 | 11.6  10.7 | 11.9  10.8 | 12.3  9.9 | p<0.01^3^  p<0.01^3^ |

^1^One-way analysis of variance, ^2^Kruskal-Wallis test, ^3^Chi-square test

^4^Daily physical activity, from 1 = very little to 10 = very much.

BMI = Body Mass Index; OC = Oral Contraceptive, HRT = Hormone Replacement Therapy; IUD = Intra Uterine Device.

**Supplementary table S3.** Demographics and life style characteristics in the five different user groups of facial cream. The NOWAC study, 2003-2011, Tromsø, Norway.

|  | **Use of facial cream** | | | | | |
| --- | --- | --- | --- | --- | --- | --- |
|  | **None** | **Light** | **Moderate** | **Frequent** | **Heavy** | **Group comparison** |
| Reported frequency of facial cream use | Never | 1-4 times/month | 2-4 times/week | 5-7 times/week | ≥2 times/day |  |
| Body surface creamed/day (%) | 0 | 0.2-0.4 | 1.3 | 2.4-3.0 | 6.0 | - |
| Number of Observations | 7927 | 4352 | 4598 | 68817 | 28508 | - |
| Characteristics | Mean (SD) | Mean (SD) | Mean (SD) | Mean (SD) | Mean (SD) | p-value |
| Age at answering questionnaire | 55.0 (5.0) | 54.6 (4.8) | 54.7 (5.0) | 54.9 (4.9) | 54.7 (4.8) | p<0.01^1^ |
| Education, years | 12.2 (3.7) | 12.6 (3.7) | 12.7 (3.5) | 12.8 (3.5) | 13.5 (3.5) | p<0.01^1^ |
| Household income, 10 000’s NOK | 49.3 (22.2) | 50.9 (21.8) | 52.0 (21.3) | 56.8 (21.6) | 60.4 (22.1) | p<0.01^1^ |
| BMI, kg/m^2^ | 27.2 (5.4) | 27.0 (5.0) | 26.5 (4.7) | 25.2 (4.0) | 24.2 (3.5) | p<0.01^1^ |
| Physical activity^4^ | 5.4 (2.0) | 5.4 (1.9) | 5.5 (1.9) | 5.9 (1.8) | 6.1 (1.8) | p<0.01^1^ |
| Age at menarche | 13.2 (1.5) | 13.2 (1.5) | 13.3 (1.4) | 13.3 (1.4) | 13.3 (1.4) | p<0.01^1^ |
| Age at menopause | 48.7 (5.1) | 48.6 (5.1) | 49.0 (4.8) | 49.0 (4.8) | 49.2 (4.8) | p<0.01^1^ |
| Age at first birth | 23.9 (4.6) | 24.0 (4.7) | 24.0 (4.6) | 24.0 (4.5) | 24.5 (4.6) | p<0.01^1^ |
| Number of children | 2.4 (1.4) | 2.3 (1.3) | 2.3 (1.3) | 2.2 (1.1) | 2.0 (1.1) | p<0.01^1^ |
|  | Median  (25^th^ – 75^th^ percentile) | Median  (25^th^ – 75^th^ percentile) | Median  (25^th^ – 75^th^ percentile) | Median  (25^th^ – 75^th^ percentile) | Median  (25^th^ – 75^th^ percentile) |  |
| Breastfeeding, months | 11 (4- 21) | 12 (5-22) | 12 (5-21) | 11 (5-19) | 11 (6-19) | p<0.01^2^ |
| Alcohol, gr/day | 1.0 (0-3.6) | 1.5 (0-4.2) | 1.6 (0.4-4.9) | 2.4 (0.9-5.9) | 3.8 (1.2-7.4) | p<0.01^2^ |
|  | % | % | % | % | % |  |
| Smoking status  Never  Former  Current | 41.6  30.0  28.4 | 39.9  31.4  28.7 | 39.9  33.5  26.6 | 35.9  40.3  23.9 | 34.3  44.1  21.6 | p<0.01^3^ |
| Use of OC  Never  Former  Current | 48.2  51.2  0.5 | 44.8  54.7  0.5 | 43.7  55.8  0.5 | 39.2  60.2  0.5 | 33.4  66.1  0.6 | p<0.01^3^ |
| Use of HRT  Never  Former  Current | 71.8  18.0  10.2 | 71.2  18.9  9.9 | 70.7  18.6  10.7 | 62.3  22.4  15.3 | 56.0  24.4  19.6 | p<0.01^3^ |
| Use of Hormone IUD  Ever  Current | 9.2  7.8 | 10.2  9.2 | 10.7  10.3 | 11.4  10.8 | 12.3  11.3 | p<0.01^3^  p<0.01^3^ |

^1^One-way analysis of variance, ^2^Kruskal-Wallis test, ^3^Chi-square test

^4^Daily physical activity, from 1 = very little to 10 = very much.

BMI = Body Mass Index; OC = Oral Contraceptive, HRT = Hormone Replacement Therapy; IUD = Intra Uterine Device.

**Supplementary table S4.** Demographics and life style characteristics in the five different user groups of hand cream. The NOWAC study, 2003-2011, Tromsø, Norway.

|  | **Use of hand cream** | | | | | |
| --- | --- | --- | --- | --- | --- | --- |
|  | **None** | **Light** | **Moderate** | **Frequent** | **Heavy** | **Group comparison** |
| Reported frequency of hand cream use | Never | 1-4 times/month | 2-4 times/week | 5-7 times/week | ≥2 times/day |  |
| Body surface creamed/day (%) | 0 | 0.4-0.8 | 2.6 | 4.8-6.0 | 12.0 | - |
| Number of Observations | 16629 | 16363 | 15707 | 33663 | 31840 | - |
| Characteristics | Mean (SD) | Mean (SD) | Mean (SD) | Mean (SD) | Mean (SD) | p-value |
| Age at answering questionnaire | 54.7 (4.9) | 54.5 (4.8) | 54.6 (4.8) | 55.0 (4.9) | 54.9 (4.8) | p<0.01^1^ |
| Education, years | 12.7 (3.6) | 13.3 (3.6) | 13.0 (3.5) | 12.8 (3.5) | 12.9 (3.3) | p<0.01^1^ |
| Household income, 10 000’s NOK | 55.0 (22.7) | 58.3 (22.1) | 58.1 (21.7) | 56.4 (22.1) | 56.5 (21.5) | p<0.01^1^ |
| BMI, kg/m^2^ | 25.8 (4.6) | 25.6 (4.3) | 25.3 (4.1) | 25.1 (4.0) | 24.8 (3.9) | p<0.01^1^ |
| Physical activity^4^ | 5.6 (2.0) | 5.6 (1.8) | 5.8 (1.8) | 6.0 (1.8) | 6.1 (1.8) | p<0.01^1^ |
| Age at menarche | 13.3 (1.4) | 13.3 (1.4) | 13.3 (1.4) | 13.3 (1.4) | 13.2 (1.4) | p<0.01^1^ |
| Age at menopause | 48.9 (4.9) | 49.0 (4.7) | 49.2 (4.6) | 49.0 (4.9) | 49.0 (4.9) | p<0.01^1^ |
| Age at first birth | 23.9 (4.6) | 24.4 (4.6) | 24.2 (4.5) | 24.1 (4.5) | 24.1 (4.5) | p<0.01^1^ |
| Number of children | 2.2 (1.2) | 2.2 (1.2) | 2.2 (1.1) | 2.2 (1.2) | 2.1 (1.1) | p<0.01^1^ |
|  | Median  (25^th^ – 75^th^ percentile) | Median  (25^th^ – 75^th^ percentile) | Median  (25^th^ – 75^th^ percentile) | Median  (25^th^ – 75^th^ percentile) | Median  (25^th^ – 75^th^ percentile) |  |
| Breastfeeding, months | 11 (4- 19) | 12 (6-21) | 12 (6-20) | 11 (5-19) | 11 (5-19) | p<0.01^2^ |
| Alcohol, gr/day | 2.1 (0.5-5.7) | 2.4 (0.9-6.2) | 2.5 (0.9-6.3) | 2.4 (0.9-5.9) | 2.4 (0.9-6.0) | p=0.10^2^ |
|  | % | % | % | % | % |  |
| Smoking status  Never  Former  Current | 32.9  37.4  29.7 | 37.6  39.6  22.8 | 37.4  40.0  22.6 | 37.2  39.7  23.1 | 35.5  41.5  23.0 | p<0.01^3^ |
| Use of OC  Never  Former  Current | 41.2  58.3  0.5 | 38.4  61.1  0.5 | 38.7  60.7  0.6 | 39.2  60.3  0.5 | 37.3  62.2  0.5 | p<0.01^3^ |
| Use of HRT  Never  Former  Current | 66.0  21.0  13.0 | 65.9  20.5  13.6 | 63.0  22.4  14.6 | 60.9  22.7  16.4 | 58.8  23.5  17.7 | p<0.01^3^ |
| Use of Hormone IUD  Ever  Current | 10.9  10.1 | 12.2  11.9 | 11.9  11.4 | 11.2  10.1 | 11.3  10.3 | p<0.01^3^  p<0.01^3^ |

^1^One-way analysis of variance, ^2^Kruskal-Wallis test, ^3^Chi-square test

^4^Daily physical activity, from 1 = very little to 10 = very much.

BMI = Body Mass Index; OC = Oral Contraceptive, HRT = Hormone Replacement Therapy; IUD = Intra Uterine Device.

**Figure S.1**. Distribution of the calculated total percentage of body surface creamed per day (n=114 202). The NOWAC study, 2003-2011, Tromsø, Norway.


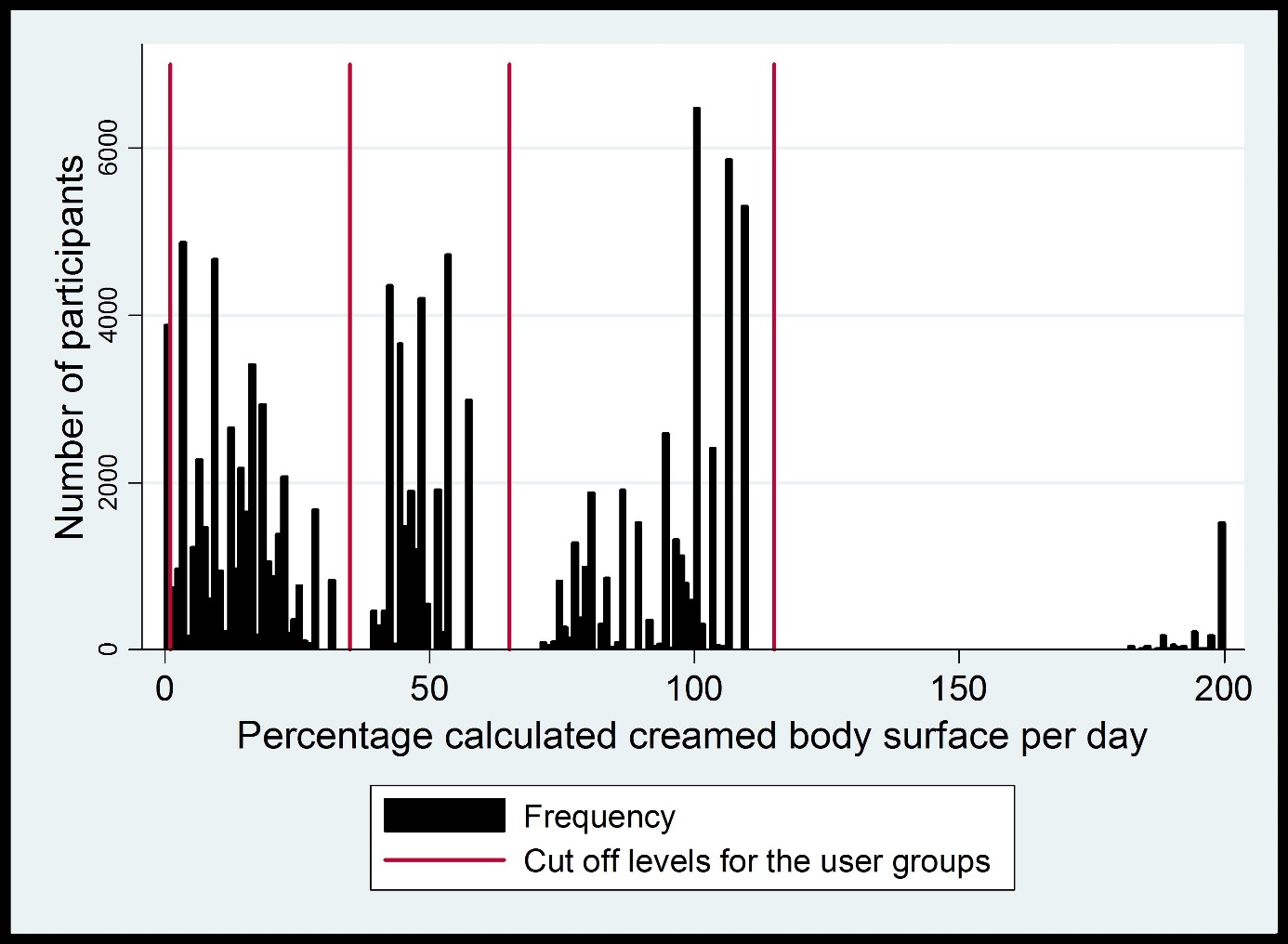

Supplement: Additional file 1: Table S1. — Conversion table of type and frequency of skin care product into percentage body area creamed per day. The NOWAC study, 2003–2011, Tromsø, Norway. Table S2. Demographics and life style characteristics in the five different user groups of body lotion. The NOWAC study, 2003–2011, Tromsø, Norway. Table S3. Demographics and life style characteristics in the five different user groups of facial cream. The NOWAC study, 2003–2011, Tromsø, Norway. Table S4. Demographics and life style characteristics in the five different user groups of hand cream. The NOWAC study, 2003–2011, Tromsø, Norway. Figure S1. Distribution of the calculated total percentage of body surface creamed per day (n = 114 202). The NOWAC study, 2003–2011, Tromsø, Norway. (DOCX 208 kb) [file 13690_2016_165_MOESM1_ESM.docx]
